# How much of Virus-Specific CD8 T Cell Reactivity is Detected with a Peptide Pool when Compared to Individual Peptides?

**DOI:** 10.3390/v4112636

**Published:** 2012-10-29

**Authors:** Wenji Zhang, Ioana Moldovan, Oleg S. Targoni, Ramu A. Subbramanian, Paul V. Lehmann

**Affiliations:** Cellular Technology Limited, Shaker Heights, Ohio 44122; Email: Wenji.Zhang@immunospot.com (W.Z.), Ioana.Moldovan@immunospot.com (I.M.), Oleg.Targoni@immunospot.com (O.S.T.), Paul.Lehmann@immunospot.com (P.V.L.)

**Keywords:** CEF, avidity, ELISPOT, immune monitoring, vaccine, PBMC

## Abstract

Immune monitoring of T cell responses increasingly relies on the use of peptide pools. Peptides, when restricted by the same HLA allele, and presented from within the same peptide pool, can compete for HLA binding sites. What impact such competition has on functional T cell stimulation, however, is not clear. Using a model peptide pool that is comprised of 32 well-defined viral epitopes from Cytomegalovirus, Epstein-Barr virus, and Influenza viruses (CEF peptide pool), we assessed peptide competition in PBMC from 42 human subjects. The magnitude of the peptide pool-elicited CD8 T cell responses was a mean 79% and a median 77% of the sum of the CD8 T cell responses elicited by the individual peptides. Therefore, while the effect of peptide competition was evident, it was of a relatively minor magnitude. By studying the dose-response curves for individual CEF peptides, we show that several of these peptides are present in the CEF-pool at concentrations that are orders of magnitude in excess of what is needed for the activation threshold of the CD8 T cells. The presence of such T cells with very high functional avidity for the viral antigens can explain why the effect of peptide competition is relatively minor within the CEF-pool.

## 1. Introduction

The CD8 T cell system has evolved to recognize antigens that are actively biosynthesized in cells. During antigen presentation, short peptides derived from such intracellular antigens are generated through active processing pathways and are loaded onto nascent HLA-Class I molecules for recognition by T cells [1]. These short peptides bind within a biding pocket created by the alpha 2 and alpha 3 domains of the Class I molecule, capable of accommodating approximately nine amino acids long peptides. While a preponderance of Class I epitopes are nanomers [2,3], natural Class I peptides ranging from 8 to 12 amino acids in length have been described [4,5,6]. Extracellular protein antigens are generally endocytosed by the antigen presenting cells, actively degraded, and presented on Class II molecules to CD4 cells [7]. These basic rules of antigen presentation permit CD8 T cells to identify and destroy cells that are actively infected while sparing bystander cells that may have acquired the antigen without themselves being infected.

Soluble antigenic peptides of the right length and sequence can directly bind to Class I molecules located on the surface of cells. This (highly artificial) way of presenting antigen to the CD8 T cell is at the very heart of immune monitoring when the infectious agent itself cannot be introduced into the assay system utilizing the natural endogenous Class I presentation pathway. While CD4+ T cell recognition in immunoassay contexts can be accomplished by providing full-length protein antigens extracellularly which can then be endocytosed and processed by antigen presenting cells (APC) and presented to CD4 cells, for CD8 T cell recognition, immune assays require short peptides.

Initially, experiments performed with inbred mice (that are homozygotes for MHC Class I alleles) utilizing simple antigenic systems suggested that CD8 T cells recognize few peptides of antigens as immune dominant determinants [8,9]. Such highly focused CD8 T cell recognition, however, seems to be more an exception in the outbred human population’s response to complex infectious organisms. Each HLA Class I allele has a unique peptide-binding grove that defines the binding specificity of the allele. With a multitude of codominant HLA Class I alleles possible at each locus, each human subject expresses a unique set of HLA-alleles that recognize a unique set of antigenic peptides that the individual can present to CD8 T cells. Such individualized antigen presentation is thought to have evolved under strong evolutionary pressure to prevent antigenic escape: even if an infectious agent mutates an epitope and evades immune recognition in one individual, the same mutation will not preclude immune recognition in other individuals who express a different set of Class I molecules, and therefore, recognize a different set of antigenic epitopes [10].

Because of the highly unique nature of epitope recognition within any given individual, the use of larger peptide pools has become mainstream for immune monitoring purposes. This approach of offering a multitude of putative antigen-derived epitopes for CD8 T cell recognition leaves it up to the test subject’s specific HLA-alleles to bind and present those few relevant peptides that are present in the peptide pool capable of stimulating cognate T cells present within the subject’s repertoire.

While utilizing peptide pools is a convenient approach for immune monitoring, it is currently unknown to what extent individual peptides in a pool interfere with each other in CD8 T cell assays. For example, it is conceivable that different peptides within the pool compete for binding to individual HLA alleles, and peptides that have higher binding affinity for that allele could outcompete those with lower affinity [11]. In order for extracellular peptides to bind to a Class I molecule on the surface of an APC, they need to displace peptides that had been loaded intracellularly. Even in a hypothetical scenario where five peptides have the same binding affinity for an HLA allele (e.g., HLA-A2-0201), each peptide will compete with the other peptides for binding, thereby reducing the number of bound peptides of any given type to one-fifth of what would have bound had the peptide been presented in isolation. Peptides with higher affinity will stably occupy more HLA molecules and are likely to be presented preferentially when compared to peptides of lower affinity. Since peptide competition is bound to occur when working with peptide pools, the magnitude of responses elicited by any given constituent peptide can be expected to be lower in the context of the pool than when it is assessed singly.

While the use of peptide pools is a necessity for T cell diagnostics given the limited numbers of PBMC available for most of clinical samples and the time required to assess peptide-specific responses individually, how much of the antigen-specific CD8 T cell reactivity goes undetected when peptide pools are used remains unclear. We addressed this issue in a cohort of 42 normal subjects by comparing the magnitude of responses elicited by CEF peptide pool and the 32 peptides that comprise this pool singly. The CEF peptide pool, originally described by Currier and coworkers, is comprised of well-defined peptides derived from Cytomegalovirus (C), Epstein Bar virus (E) and Flu viruses (F) [12] and is widely used as a positive control for CD8 T cell activation. Our study of this highly characterized peptide pool model can provide insights into the validity of using peptide pools in less defined antigenic systems.

## 2. Results and Discussion

### 2.1 Individual Donor’s CD8 T cell Responses to Individual CEF Peptides

Table 1 lists the 32 peptides that constitute the CEF-pool, their antigenic source, and the amino acid sequence of the epitopes and the originally described restricting HLA Class I allele [12]. PBMC from 42 high resolution HLA-typed healthy donors were stimulated with the 32 CEF peptides either individually or as a pool, and the number of peptide-specific CD8 T cells was measured in an IFN-γ ELISPOT assay. For both individual and pooled stimulations, the peptides were utilized at 1 μg/ml final concentration (each) and were tested in three replicate wells. Similarly, three replicate wells containing medium alone were used to establish baseline cytokine production in the absence of any peptide. Media-only control wells in these experiments routinely had ≤10 spot forming units (SFU) per well.

**Table 1:** List of CEF-pool peptides and their restricting MHC Class I alleles

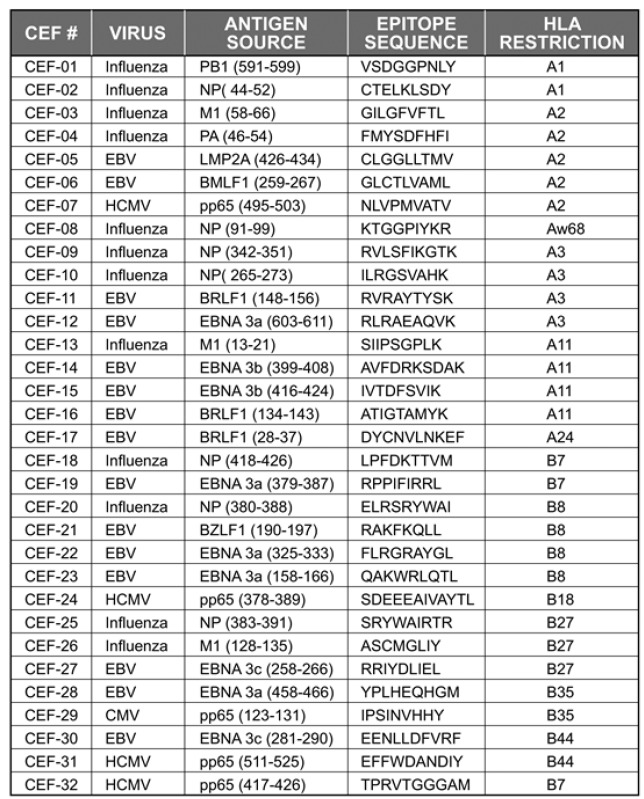

**Figure 1:**
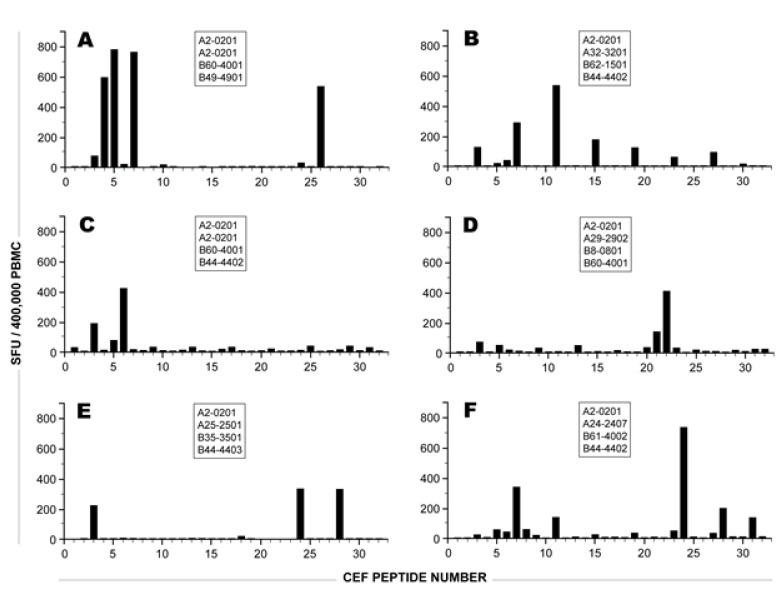
IFN-γ SFU induced by the individual CEF peptides in six HLA-A2-0201 positive donors. The other HLA-A and B alleles expressed by each of these donors are specified in the respective panels. The numbers on the X axis specify the CEF peptide number as defined in Table 1. The bars show the mean number of SFU elicited by each CEF peptide in an IFN-γ ELISPOT assay at 1 μg/ml final concentration, established in triplicate wells. The SD for each peptide-induced response was less than 20%, and the medium control was less than 10 SFU.

All donors in Figure 1, being HLA-A2-0201 positive, responded against one or more of the five HLA-A2-restricted peptides (CEF 3-7, see Table 1). However, none of the donors responded to all five HLA-A2 peptides simultaneously, and in several donors these HLA-A2 restricted peptides elicited CD8 T cells in rather low frequency. This is the case, for example, with the donor depicted in Figure 1B, who responded weakly to the HLA-A2-restricted EBV peptides CEF-5 and CEF-6; and moderately to the HLA-A2 restricted CEF-7. The dominant recall response in this subject was elicited rather by an HLA-A3-restricted EBV peptide, CEF-11 (Table 1), though this donor did not express this allele. Similarly, as depicted in Figure 1F, peptide CEF-24, an HCMV peptide that was originally described as an HLA-B18-restricted epitope, elicited a stronger recall response than the HLA-A2-restricted HCMV peptide CEF-7, though this donor was HLA-B18-negative, but HLA-A2-positive. In general, while responses elicited against predicted epitopes constituted a fraction of the recall responses, frequently, unpredicted reactivities were seen. This is in agreement with studies that document the ability of a large number of peptides with known HLA binding specificities to bind to two or more previously unpredicted HLA alleles [13]. In fact, increasing evidence suggests that such promiscuity within the HLA Class I antigenic system is rather extensive [14,15,16].

The data in Figure 1 also illustrates the level of variability with which the individual donors respond to individual peptides. There was no peptide that was prevalently recognized across the 42 donors tested. Therefore, the data illustrates the need for working with peptide pools and substantiates the notion that individual peptides might not provide reliable information on the magnitude and diversity of the CD8 T cell responses raised against complex antigenic systems including viruses.

### 2.2 The Sum of CD8 T Cells Responding to Individual CEF Peptides Approximates the Number of CD8 T Cells Triggered by the CEF-Pool

The primary hypothesis behind these experiments is that the individual peptides in a peptide pool restricted by the same HLA allele compete for binding to that allele, and therefore, the sum of CD8 T cells stimulated by the individual peptides are higher than the number of CD8 T cells stimulated by the pool of these peptides.

**Table 2:** Number of IFN-γ SFU elicited by the CEF-pool (32 peptides in a single stimulation) *vs*. the sum of IFN-γ SFU elicited by the individual 32 CEF peptides.

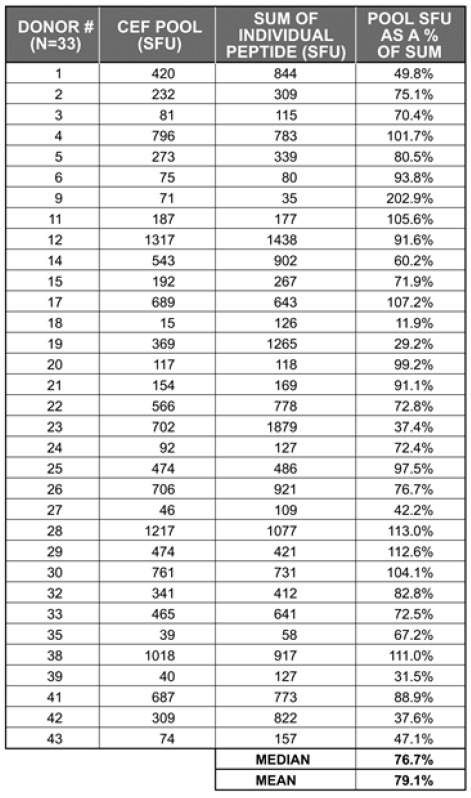

To establish the extent of peptide competition within the CEF-pool, we tested 42 donors for the CEF-pool-elicited recall response (all 32 peptides present in a single stimulation, each peptide at 1 µg/ml) and the sum of IFN-γ SFU elicited by the individual peptides. To avoid inter-assay variations, the peptide pool and the individual peptides were tested in parallel in the same experiment for each donor. Table 2 summarizes the results for the 33 donors in which the CEF peptide pool induced a recall response ≥ 2 fold the media-only control responses. The table shows what percentage of the CD8 recall response is elicited by the CEF peptide pool *vs.* the sum of SFU induced by the individual peptides (the latter defining 100%). In 17 of these 33 head-to-head comparisons, the responses to the peptide pool was reduced by >20%. In all, the pool generated a median of 77% and a mean of 79% of the responses observed when the peptides were tested individually.

While the data in Table 2 provides clear evidence for the peptide pool eliciting lower recall responses than the sum of the individual peptides, the magnitude of reduction was small. Therefore, given our inability to reliably predict individual antigenic peptides recognized by individual donors within a cohort (Figure 1), and finite sample availability in typical clinical studies, these data suggest that working with peptide pools offers a necessary and valid approach to assessing immune responses in outbred populations.

### 2.3 Functional Avidity of CEF Peptide-Specific T Cells.

We next set out to test the hypothesis that the relatively minor peptide competition we observe with the use of the CEF-pool is the result of high avidity CD8 T cell recognition of the individual peptides contained in the pool. Affinity describes the strength of binding between two molecules in a monovalent interaction (e.g., between the F(ab) fragment of an antibody and an antigen). The binding affinity of a peptide for the MHC molecule and the stability of the MHC-peptide complex have the potential to affect T cell activation. However, in addition to the MHC-peptide interactions, effective T cell activation depends on additional complex molecular interactions at the immunological synapse. The Affinity of the T cell receptor (TCR) for the MHC-peptide complex on the APC does not directly describe the extent to which the APC will stimulate the T cell; rather, a high TCR off-rate and serial triggering is known to be more stimulatory to the T cell than the strong binding of TCR to the MHC-peptide complex [17,18]. In addition to the interaction of the TCR with the MHC-peptide complexes, several other costimulatory molecules on the surface of both cell types (APC and the T cell) are involved in setting the T cell’s activation threshold [19,20]. In biochemical terms, one would define such multivalent interactions by the term avidity. However, because even molecular avidity does not account for the complexity of T cell signaling and eventual T cell activation, the latter is best defined as functional avidity, which is expressed as the antigen/peptide concentration that causes 50% maximal T cell activation (K_eff_ value) [21,22].

If, therefore, CEF peptides are recognized by CD8 T cells with high functional avidity, then the 1 μg/ml peptide concentration that is commonly used in immune assays may be in excess of what is needed to induce maximal T cell stimulation. When the density of MHC-nominal peptide on the APC (the peptide for which the T cell is specific) exceeds what is needed for T cell activation, the exchange of excess nominal peptide by a competing peptide will have no effect on T cell activation. What is, therefore, the functional avidity of T cells that recognize a given CEF peptide?

**Figure 2:**
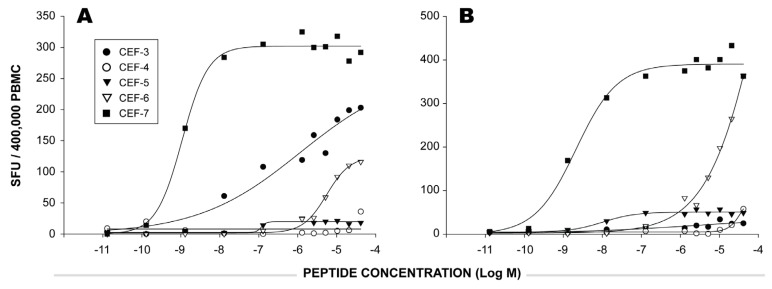
The functional avidity of CEF peptide-reactive CD8 T cells as exemplified in two donors. In each panel depicts one HLA-A2-0201 positive donor. The PBMC were tested in an IFN-γ ELISPOT assay with decreasing concentrations of five HLA-A2 restricted CEF peptides (CEF 3, 4, 5, 6, 7, as specified by the symbols). Each data point represents SFU from a single well. For each donor the SFU were plotted on the Y axis against the concentration of peptides used for stimulation in the X axis.

Figure 2 shows the IFN-γ ELISPOT responses elicited in two HLA-A2-0201 positive PBMC donors by different concentrations of the five HLA-A2 restricted peptides contained in the CEF-pool (CEF 3, 4, 5, 6, 7). In both donors, peptide CEF-7 elicited CD8 T cells of high frequency that also had a high functional avidity: a concentration of 10^-9.5^ M of the peptide sufficed to activate 50% of these T cells (K_eff_ 50 = 10^-9.5^ M). In contrast, CEF-6-reactive CD8 T cells from the donor depicted in Figure 2B were of high frequency, but of low avidity. Also, in the donor depicted in Figure 2A, the CEF-6 recall response is of low functional avidity, but occurs at a lower frequency.

The data presented in Figure 2 shows that the individual HLA-A2 restricted CEF peptides are recognized by CD8 T cells of highly different functional avidities in HLA-A2 positive donors. For some of these peptides, as little as 10^-9^ M peptide was sufficient to induce 50% T cell activation. For such T cells, the 1 µg peptide concentration (that is commonly used in immune assays) is orders of magnitude in excess of what is required to stimulate them. For other peptides, the 1 µg/ml concentration is not yet sufficient to activate all peptide-reactive T cells, as was the case for the CEF-4- and CEF-6-reactive T cells in these two donors.

Based on such data, one might expect that peptide competition in the context of peptide pools will selectively affect T cells with low, but not high functional avidity. In these two donors, the CEF-7 peptide response is unlikely to be inhibited by peptide competition. Since this peptide present in the CEF peptide pool is orders of magnitude in excess, reducing CEF-7 peptide concentration on the surface of the APC by other peptides that have HLA-A2-binding properties will not readily impact the activation threshold of CEF-7-specific CD8 T cells. For the CEF-4 and CEF-6-specific CD8 T cells, however, even relatively small reductions in the numbers of peptide-HLA-A2 complexes on the APC will have a major impact on the numbers of T cells activated.

**Figure 3:**
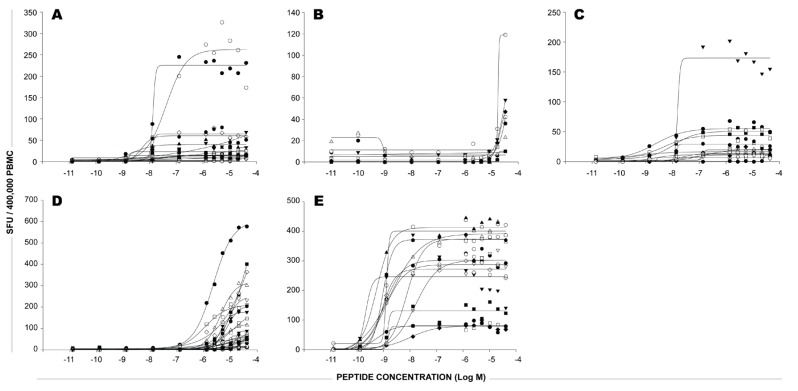
The functional avidity of CEF peptide-reactive CD8 T cells in our HLA-A2-0201-positive donor cohort. In each panel, data are shown for one of the five HLA-A2 restricted CEF peptides (CEF 3, 4, 5, 6, and 7, in panels A-E, respectively). The different HLA-A2-positive donors are specified by symbols. The PBMC were tested in an IFN-γ ELISPOT assay with decreasing concentrations of the HLA-A2 peptides. Each data point represents SFU from a single well. For each donor and peptide tested, the SFU were plotted on the Y axis against the concentration of peptides used for stimulation in the X axis.

The data from both donors above show that there are low avidity T cell responses to CEF (that should be sensitive to peptide competition) and high avidity T cell responses (that should not be sensitive to peptide competition). Because the functional avidities with which the five individual HLA-A2-restricted peptides were recognized by CD8 T cells in these two donors were orders of magnitudes apart (providing data with fundamentally different implications for peptide competition), we tested how representative the above two donors are. If these donors are representative of the human population, we should see a similar pattern in our larger cohort. To explore this further, dose response curves for the five HLA-A2 restricted CEF peptides were evaluated among the HLA-A2-positive donors in our cohort. Each panel in Figure 3 shows the dose response curve to one of the five CEF peptides.

The maximal numbers of CD8 T cells stimulated by each of the five HLA-A2-restricted peptides showed considerable donor-dependent variation signifying the frequency of peptide-specific CD8 T cells within the PBMC assayed. However, frequently, peptides that were recognized by CD8 T cells with high functional avidity in one donor were also recognized by high avidity T cells in other donors (CEF-7) and the same applied for peptides recognized by low avidity T cells (CEF-4 and CEF-6).

The systematic avidity testing of this cohort of subjects suggests that most donors possess high avidity T cells that recognize one or more CEF peptides. Although not providing direct proof, the data strongly suggests that such high avidity responses contribute dominantly to the overall pooled responses. Should this hypothesis be true, the high avidity CEF-7 responses (being a dominant contributor) would correlate with the CEF-pool responses while low avidity CEF-4 responses may not. Using a cohort of 28 HLA-A2 subjects in our cohort, we evaluated the statistical relationship between the CEF-4 or the CEF-7 individual peptide responses and the CEF-pool responses. While we found no correlation between the low avidity CEF-4 responses and the CEF-pool responses in these 28 donors, we found highly significant statistical correlation between the high avidity CEF-7 responses and the CEF-pool responses (Pearson r=0.7220; p<0.0001). An alternate nonparametric statistical analysis also found significant correlation between CEF-pool responses and CEF-7 responses (Spearman r=0.7286; p<0.0001) but not CEF-4 responses. Hence, while higher CEF-7 responses generally resulted in higher CEF-pool responses among our HLA-A2 donors, no such relationship existed between low avidity CEF-4 responses and the CEF-pool responses among these subjects. Thus, the presence of such high avidity CEF peptide-reactive T cells can potentially explain why peptide competition is not more pronounced than what is observed in this study.

In fact, many of the epitopes originally selected by Currier and coworkers for the CEF peptide pool [12] are well-known dominant epitopes from influenza viruses [23]; and the herpes viruses, CMV and EBV [24]. An additional reason for the high avidity of these epitopes could be the nature of the antigenic source of these peptides: specifically, two viruses that chronically infect humans (EBV and CMV), and one acute virus that infects humans repeatedly over their lifetime (influenza). CMV seroprevalence is reported to be at nearly 60% by six years of age and nearly 91% by 80 years of age [25] and EBV prevalence was at nearly 95% of the adults screened [26]. Similarly, prevalence of influenza A antibodies reached nearly 100% by the age of 12 years in a large cohort of 1,111 children and adolescents [27]. Both the chronic infection with multiple reactivations (CMV and EBV) and repeated acute infection (influenza) scenarios could favor repeated stimulation of T cell populations resulting in high avidity clones emerging as dominant lineages within individuals.

As opposed to such viral antigen-reactive T cells (*i.e.* “foreign”), autoantigen-reactive T cells undergo negative selection in the thymus [28]. For example, when an autoantigen of the brain, myelin basic protein (MBP)-specific T cell repertoire was compared in mice that were MBP knock-out (for these T cells MBP is a foreign antigen), or were of the congenic wild-type strain (for which MBP is a self-antigen), we found that the T cells in the KO mice recognized MBP with a orders of magnitude higher functional avidity that the MBP-specific T cells in the wild type mice [29,30]. One can assume that also tumor antigens, being self-antigens, follow similar rules, and are recognized by low avidity T cells. Furthermore, during chronic infections, antigen-specific T cells can undergo exhaustion leaving a repertoire of low functional avidity cells. In such situations, peptide competition might have a larger impact on the detection of the antigen specific T cells using peptide pools compared to what we observed in the viral CEF system.

## 3. Experimental Section

### 3.1. Thawing and Handling of Cryopreserved PBMC and HLA Typing

Cryopreserved PBMC from 42 healthy human donors were acquired from a library of characterized frozen PBMC (ePBMC, CTL, Shaker Heights, Ohio). High resolution HLA typing of the PBMC was performed using SSP methodology. PBMC were thawed following a protocol that we have established to provide the optimal recovery and functionality for cryopreserved PBMC [31]. Following the thaw and wash procedures, PBMC were resuspended at a final concentration of 4 X 10^6^ PBMC/ml in CTL-Test Medium (CTLT-005) of which 100 µl (400,000 cells) was plated per well into the ELISPOT assay. The thawed PBMC were not “rested” before testing, but were plated directly into the assay as resting does not significantly improve the performance of these PBMC [32].

### 3.2 CEF Peptides

The CEF peptide pool and the 32 constituent peptides were acquired from CTL (CTL-CEF-002). Peptides were of >95% purity. Each of the individual CEF peptides were dissolved at 2 µg/ml in CTL-Test Medium of which 100 µl was plated per well, resulting in a final concentration of 1 µl/ml. In experiments that aimed at establishing the dose response curve to individual peptides, the peptides were plated in serial dilution, as specified in the respective Figures. In the CEF peptide pool solution each of the peptides were at 2 µg/ml, also dissolved in CTL-Test Medium, and plated into the assay at 1 µg/ml final concentration per peptide.

### 3.3. Human Interferon-γ ELISPOT Assay

The human interferon-γ ImmunoSpot kit (CTL-HIFNG-1/5M) was acquired from CTL. The assay was performed according to the manufacturer’s recommendations. The PVDF membranes were not pre-wetted with ethanol as it is not required nor recommended for this kit. The individual CEF peptides, the CEF peptide pool, or medium alone for the negative control were plated into the ELISPOT assay plates that had been pre-coated with the IFN-γ capture antibody. The plates containing the peptides and medium control were stored at 37^o^C in a CO_2_ incubator until the cells were ready for plating. The thawed PBMC were added at 400,000 cells/well using wide-bore pipette tips. Plates were gently tapped on each side to ensure even distribution of the cells as they settle, and incubated for 24 hours at 37^o^C in a CO_2_ incubator. Following completion of the ELISPOT assay, the plates were air dried in a laminar flow hood prior to analysis.

ELISPOT plates were scanned and analyzed using an ImmunoSpot S6 Core Reader by CTL. Spot Forming Units (SFU) were automatically calculated by the ImmunoSpot Software for each antigen stimulation condition and the medium (negative) control using the SmartCount™ and Autogate™ functions [33]. Peptides that generated at least twice the spot forming units (SFU) over the control wells lacking any peptide (media-only stimulation) were defined as eliciting a positive response. When media-only wells lacked any spots, as was the case for several PBMC, these control wells were arbitrarily given a SFU value of two spots, to avoid division by zero. Data are presented as mean SFU induced by the specified peptide or the CEF-pool minus the SFU count in the negative control.

## 4. Conclusions

Peptides compete for binding to MHC molecules and occupancy of the restricting MHC allele by an irrelevant peptide can interfere with the stimulatory potential of T cells that are specific for the cognate peptide. In this study, however, inhibitory effects due to peptide competition were found to be relatively low for the CEF peptide pool; our data suggests this is due to the high functional avidity of T cells recognizing several CEF peptides. Our data also shows that some peptides within the CEF-pool are of lower avidity and therefore more susceptible to peptide competition (such as CEF-4 in Figure 3). While peptide competition could theoretically affect T cells with low functional avidity for a given peptide, such as tumor- or autoantigen-specific T cells, it did not have a major impact on immune responses directed against the viral peptides that constitute the CEF peptide pool.
